# Novel Algorithm for Monogenic Noninvasive Prenatal Testing With Highly Similar Parental Pathogenic Haplotypes: A Representative Case of Congenital Adrenal Hyperplasia Pedigree

**DOI:** 10.1155/humu/9990873

**Published:** 2025-10-13

**Authors:** Wenjing Zhou, Fulin Liu, Shaojun Li, Di Wu, Jiyun Yang

**Affiliations:** ^1^Sichuan Provincial Key Laboratory for Human Disease Gene Study, Center of Medical Genetics, Sichuan Provincial People's Hospital, School of Medicine, University of Electronic Science and Technology of China, Chengdu, China; ^2^Celula (China) Medical Technology Co., Ltd., Chengdu, China; ^3^Research Unit for Blindness Prevention of Chinese Academy of Medical Sciences (2019RU026), Sichuan Academy of Medical Sciences, Chengdu, China

**Keywords:** algorithmic improvement, bioinformatics, congenital adrenal hyperplasia, genetic diagnosis, noninvasive prenatal testing, relative haplotype dosage analysis

## Abstract

Noninvasive prenatal testing (NIPT) has been widely used in various monogenic recessive disorders based on relative haplotype dosage (RHDO) analysis. We accepted a congenital adrenal hyperplasia (CAH) pedigree with highly similar parental pathogenic haplotypes. The initial monogenic NIPT attempt was unsuccessful due to a paucity of informative single-nucleotide polymorphisms (SNPs), prompting improvement of the current method. With a refined algorithm that deduces the fetal genotype based on dosage changes at SNPs located on a specific parental haplotype, while also effectively sidestepping allele bias introduced by hybrid capture, monogenic NIPT was successfully carried out in this family, yielding results consistent with invasive prenatal diagnosis. Theoretically, this algorithm can be employed in scenarios involving consanguineous marriages or when parents share a highly homologous haplotype, thereby broadening its applicability. Detailed methodology is described, and the advantages of our algorithm are discussed.

**Summary**

• What's already known about this topic
  o. The computation of relative haplotype dosage (RHDO) was proposed to adopt the adjacent single-nucleotide polymorphisms (SNPs) to overcome the shortage of fetal circulating cell-free DNA (cfDNA) in maternal serum with a single genomic position.
• What does this study add
  o. A novel algorithm for monogenic noninvasive prenatal testing (NIPT) was applied to bridge the gap where previous algorithms failed to analyze SNPs in consanguineous families due to a lack of combinations of parental SNPs.

## 1. Introduction

Prenatal diagnosis performed in high-risk families via invasive procedures, such as chorionic villus sampling and amniocentesis, increases the risk of miscarriage or infection and is not applicable to patients with sampling contraindications. Recently, haplotype-based NIPT has been widely used in monogenic recessive disorders as early as the seventh week of gestation [1, 2]. Fetal circulating cfDNA was applied for monogenic NIPT, while a major challenge in analyzing fetal cfDNA is that it only represents a minor fraction of maternal cfDNA. The fetal fraction (FF) varies during the course of the pregnancy but is rarely higher than 30%. However, determining whether a maternal mutation was transmitted to the fetus is more challenging since it is necessarily present in maternal cfDNA. Relative mutation dosage (RMD) was initially proposed to involve the measurement of the mutant/wild-type allelic ratio at the mutation site [3], while a major hindrance toward its routine application is the typically low abundance of cfDNA, which makes it very difficult to gather a statistically significant number of molecules from a single genomic position. To overcome this problem, adjacent SNPs are included in the computation of a “haplotype ratio,” referred to as RHDO [4]. However, its reliance on SNPs makes it difficult to apply to consanguineous families, as suitable combinations of parental SNPs can seldom be found. In the present study, we accepted a congenital adrenal hyperplasia (CAH) pedigree with highly similar parental pathogenic haplotypes in clinical practice. CAH refers to a group of autosomal recessive (AR) disorders involving monogenic defects in the various enzymes required for cortisol synthesis by the adrenal cortex [2]. More than 95% of all cases of CAH are caused by 21-hydroxylase deficiency (21-OHD, OMIM# 201910), resulting from biallelic mutations in the *cytochrome P450 family 21 subfamily A member 2* (*CYP21A2)* gene. 21-OHD is classified into three subtypes according to clinical severity: classic salt wasting, classic simple virilizing, and mild or late-onset nonclassic CAH. The classic form affects approximately 1:15,000 live births in different populations worldwide [5]. Previous algorithms failed to provide a monogenic NIPT result due to a paucity of informative SNPs, prompting improvement of the current method [6, 7]. With a new algorithm that deduces the fetal genotype based on dosage changes (DCs) at SNPs located on a specific parental haplotype, the monogenic NIPT was successfully carried out in this family. This technique is used in predicting fetal genotypes based on family linkage analysis combined with RHDO analysis in maternal cfDNA.

## 2. Case Report

**Figure 1:**
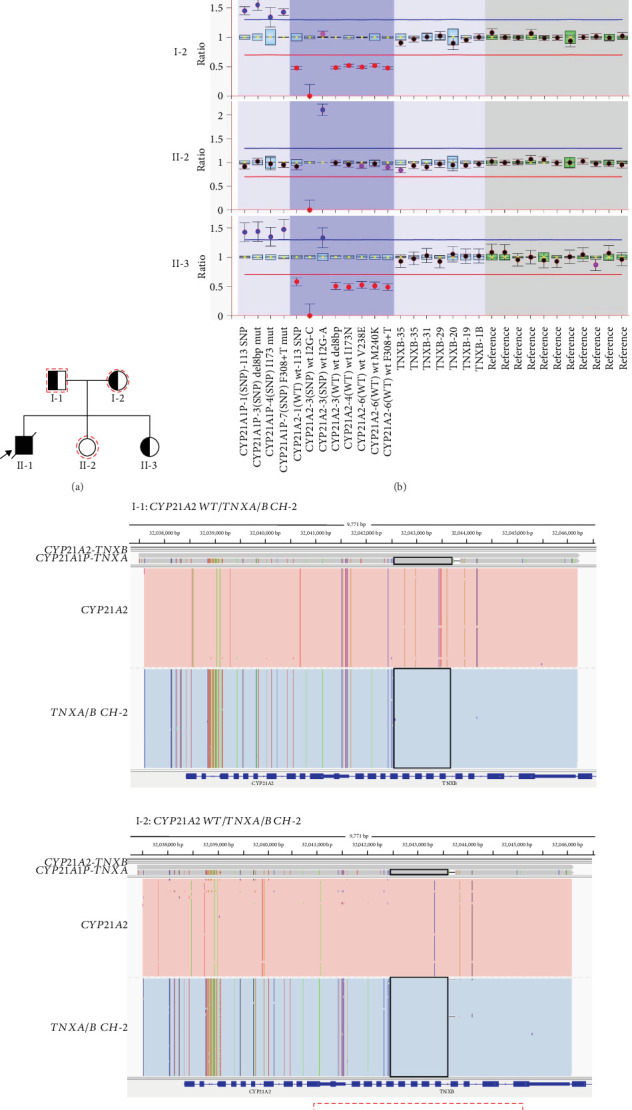
(a) Pedigree of the family with CAH. The black solid symbol and arrow indicate the index case (II-1). The parents denoted by a half-solid symbol are *CYP21A2* pathogenic variant carriers. The family members denoted with red dotted lines were used for haplotype construction. (b) MLPA testing of *CYP21A2* detected in the father (I-1), the mother (I-2), the daughter (II-2), and the fetus of the present pregnancy (II-3). (c) Long-read sequencing and schematic IGV plots revealing genotypes of the father (I-1) and the mother (I-2). The plots represent the fusion of functional genes *TNXB* and pseudogene *TNXA*, located at chr6:32,042,509–32,043,712 (GRCh38/hg38; chr:32,010,286–32,011,489 in GRCh37/hg19), resulting in a 30 kb deletion spanning exons 1–10 of *CYP21A2*. *CYP21A2: CYP21A2* without pathogenic variants. *TNXA/B CH-2*: The fusion of functional gene *TNXA* and pseudogene *TNXB*. Black box: The break interval of *TNXA/TNXB CH-2*. The deleted region denoted with a red dotted rectangle in the schematic diagram.

The family history of CAH was described in Figure 1a. The proband (II-1) was diagnosed with salt-wasting CAH and died at 33 days. The parents (I-1 and I-2) underwent next-generation sequencing (NGS) and multiplex ligation–dependent probe amplification (MLPA) tests, along with a verification of long-read sequencing (LRS) to overcome the MLPA deficiency in delineating the precise deletion boundaries (Figure 1b,c). MLPA and LRS were conducted as described in previous studies [8, 9]. In brief, primers were designed to amplify *CYP21A2*, *CYP11B1*, *CYP17A1*, *HSD3B2*, and *StAR* genes. The PCR products were purified and then quantified, followed by MLPA and constructing a library for LRS. The LRS results showed the break interval of the fusion of functional gene *TNXB* and pseudogene *TNXA*, which was located at chr6:32,042,509–32,043,712 (GRCh38/hg38; chr:32,010,286–32,011,489 in GRCh37/hg19), resulting in a 30-kb deletion spanning Exons 1–10 of *CYP21A2*.

The family had another unaffected daughter in 2017 (II-2). To clarify, the determination of the normal and mutant alleles in each parent was based on the segregation of alleles within the family and confirmed by referencing the variant's zygosity and inheritance pattern. Specifically, the unaffected child (II-2) was genotyped and found to be homozygous for the reference allele at the locus of interest, confirming that she is a noncarrier. The unaffected child was included in the analysis to serve as a control for inheritance phasing and to help resolve parental haplotypes. The genotypes provide a baseline for distinguishing between the disease-associated and nondisease-associated haplotypes, thereby improving the confidence in phasing and variant interpretation. Given that the proband was deceased and the unaffected daughter (II-2), we obtained blood samples from the pregnant mother, the father, and their unaffected daughter for monogenic NIPT during their third pregnancy in 2022. An amniotic fluid sample was simultaneously collected for invasive prenatal diagnosis.

A 213.8-kb capture panel was designed utilizing the “Selective Enrichment of Gene and Spanning SNPs” (SEGSNP) described in the study [1], which covered *CYP21A2* exons and adjacent SNPs within a 2-Mb genomic range, 203 common SNPs (minor allele frequency(MAF) > 0.45, 1000 Genomes Project Phase 3) for calculating FF, and 35 sites on the Y chromosome for fetal gender. The pregnant woman's cfDNA and fragmented gDNA of family members were subsequently captured after end-repair, barcode adapter ligation, and PCR amplification. Postcapture libraries were then subjected to PCR amplification and sequenced on the Ion Proton platform (Thermo Fisher Scientific, Lithuania). The maternal cfDNA and other family members' gDNA were assigned 5 M and 2 M reads. Sequencing reads were aligned to the human reference genome (GRCh37/hg19) using TMAP software (Version 5.2.25). Small variants of gDNA were identified with Torrent Variant Caller software (Version 5.2.25) using default parameters, after removing duplicated reads. FF was calculated by using the homologous locus in parents but with a different genotype following the equation: *ff* = 2∗(*a*/(*a* + *b*)), where *a* is the read depth of the fetal inherited paternal allele, and *b* is the read depth of the allele shared by the fetus and mother.

**Figure 2:**
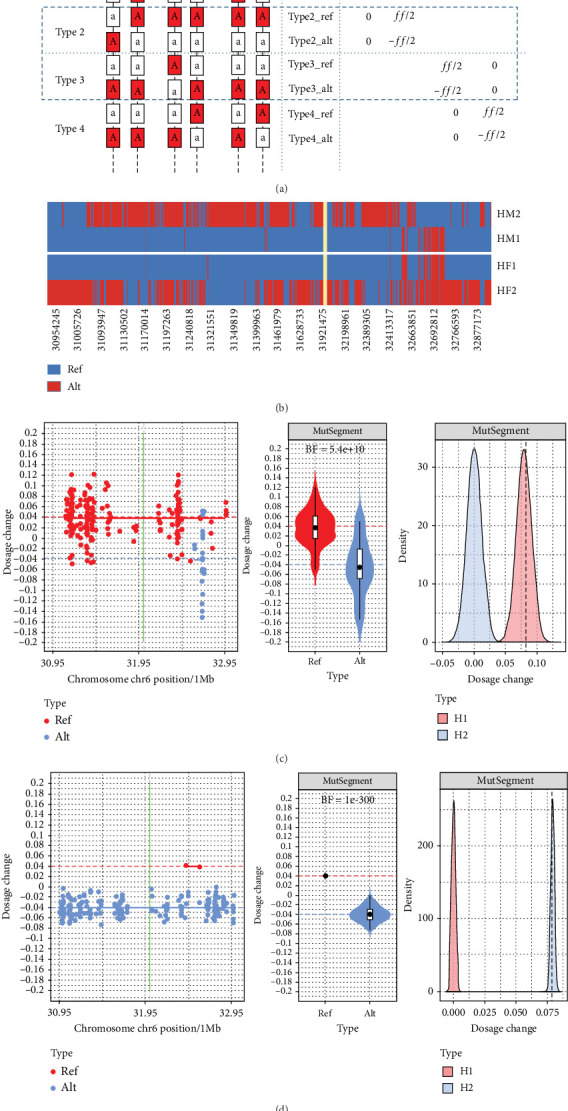
(a) Expected dosage change (DC) of ref and alt allele in the same haplotype. The red background A represents the frequency of the ref allele corresponding to the locus. The dashed blue box displays that the number of informative Type 2 and Type 3 SNPs will be extremely low when the pathogenic haplotypes are identical between parents. DC represents the expected dosage variation when a certain haplotype is inherited. When the fetus inherits the maternal pathogenic haplotype, the ref allele of type 1 goes up to *ff*/2, and the alt allele of Type 1 goes down to -*ff*/2, while the ref and alt alleles of Type 2 are both 0. When the fetus inherits the maternal wild-type haplotype, the ref allele of Type 2 goes up to *ff*/2, and the alt allele of tType 2 goes down to -*ff*/2, while the ref and alt alleles of Type 1 are both 0. The DC of the paternal haplotype follows a similar pattern to that of the maternal haplotype. (b) Schematic diagram of comparative analysis of the parental haplotypes. Blue denotes the reference (ref) allele, with red as the alternative (alt) allele. The yellow vertical line indicates where the disease-causing mutation is located. The *x*-axis represents the SNP coordinate, and from top to bottom, shows the maternal wild-type (Mhap2), the maternal pathogenic (Mhap1), the paternal pathogenic (Phap1), and paternal wild-type (Phap2) haplotypes, respectively. (c) Relative haplotype dosage (RHDO) analysis of ref and alt of maternal Type 1 haplotypes. In both the scatter plot and violin diagram, red denotes the ref allele and blue denotes the alt allele. The red dashed lines indicate the expected value of DC for ref alleles (*ff*/2) under the assumption that the fetus inherits the maternal pathogenic haplotype (Type 1), and the blue dashed lines indicate the expected value of DC for alt alleles (-*ff*/2) when presuming the fetal inheritance of Type 1. The *x*-axis in the scatter plot is the genomic coordinate, and the *y*-axis represents the DC value. Red and blue in the density plot denote the expected distribution of ref and alt alleles when presuming the fetal inheritance of the maternal pathogenic and wild-type haplotype. The dashed line represents the difference value of mean DC between the ref and alt allele of segments where pathogenic variants were located. (d) RHDO analysis of ref and alt of paternal Type 4 haplotypes. In both the scatter plot and violin diagram, red denotes the ref allele and blue denotes the alt allele. The red dashed lines indicate the expected value of DC for ref alleles (*ff*/2) under the assumption that the fetus inherits the maternal wild-type haplotype (Type 4), and the blue dashed lines indicate the expected value of DC for alt alleles (-*ff*/2) when presuming the fetal inheritance of the Type 4. Red and blue in the density plot denote the expected DC distribution when presuming the fetal inheritance of pathogenic and wild-type haplotype. The dashed line represents the difference value of the mean DC between the Type 4 ref and alt allele of segments where pathogenic variants were located.

Subsequently, an RHDO strategy was implemented [6]. Following haplotyping, the maternal haplotype harboring pathogenic variants was designated HM1, while the corresponding wild-type haplotype was labeled HM2. Similarly, paternal pathogenic and wild-type haplotypes were assigned HF1 and HF2, respectively. Informative SNPs—defined as loci heterozygous in one parent and homozygous in the other—were categorized as follows: alleles associated with HM1/HM2 were classified as Type 1/Type 2, and those linked to HF1/HF2 as Type 3/Type 4 (Figure 2a). Each of Types 1–4 represents DC when the fetus inherits maternal pathogenic haplotype (HM1), maternal wild-type haplotype (HM2), paternal pathogenic haplotype (HF1), or paternal wild-type haplotype (HF2). The Bayes factor (BF) was employed to assess the probability ratio between the two hypotheses: that is, the fetus inheriting HM1/HF1 (H1) versus HM2/HF2 (H2). The formula is BF = *p*(DC_type1/3_ − DC_type2/4_|*H*_1_) /*p*(DC_type1/3_ − DC_type2/4_|*H*_2_), where *p* indicates the probability of obtaining the current DC difference under the H1 or H2 hypothesis [1].

Initial results showed SNP counts of 227, 1, 1, and 212 for Types 1–4, respectively. Limited Type 2 and Type 3 SNPs, due to 97.97% homologous between HM1 and HF1, complicated BF computation (Figure 2b). Therefore, we modified the existing algorithm to compute the BF even with only one type of informative SNP present. This was achieved by further subdividing each informative SNP type into typeN_ref and typeN_alt, based on the reference and alternative allele (Figure 2a). With this new categorization, the counts for type1_ref, type1_alt, type4_ref, and type4_alt were 23, 204, 1, and 210, respectively. In this revised algorithm, the *p* value utilized for BF computation was no longer derived from the dosage difference between two haplotypes. Instead, it was based on the dosage difference between ref and alt sites within the same haplotype. The formula is represented as BF = *p*(DC_typeN_ref_ − DC_typeN_alt_|*H*_1_)/*p*(DC_typeN_ref_ − DC_typeN_alt_|*H*_2_), where *N* ∈ {1, 2, 3, 4}. In this context, H1 implies the fetus inherits the parental haplotype corresponding to type N, and H2 suggests the fetus does not.

The new algorithm showed a maternal type 1 BF of 5.4e+10, suggesting the fetus inherited the mother's pathogenic haplotype (Figure 2c), and a paternal type 4 BF of 1e-300, indicating the fetus inherited the father's wild-type haplotype (Figure 2d). With limited SNPs, Type 2 and Type 3 BF values were unavailable. Analysis suggested a carrier of maternal pathogenic variation for the fetus. This was later confirmed by MLPA using amniotic fluid, aligning with monogenic NIPT findings (Figure 1b). In March 2023, the mother gave birth to a healthy girl (II-3).

## 3. Case Discussion

We accepted a CAH pedigree with highly similar parental pathogenic haplotypes in clinical practice. Previous algorithms failed to provide a monogenic NIPT result due to insufficient informative SNPs, prompting the improvement of the current method. With the new algorithm deducing fetal genotype by DC at SNPs on a specific parental haplotype, the monogenic NIPT was successfully carried out in this family.

More than 95% of all cases of CAH are caused by 21-hydroxylase deficiency (21-OHD, OMIM# 201910), resulting from biallelic pathogenic variations in the *CYP21A2* gene. Prenatal diagnosis performed in high-risk families via invasive procedures, such as chorionic villus sampling and amniocentesis, increases the risk of miscarriage or infection and is inapplicable to patients with sampling contraindications. Recently, haplotype-based NIPT has been widely used in monogenic recessive disorders as early as the seventh week of gestation [1, 2, 10].

Typical RHDO-based assays classify SNPs surrounding the pathogenic variant. For instance, Type 1 SNPs are associated with HM1, while Type 2 SNPs correspond to HM2. Since fathers are always homozygous for Type 1 and Type 2 SNPs, when the fetus inherits HM1, the dosage of Type 1 SNP elevates, increasing by half of the FF. Conversely, when the fetus inherits HM2, the dosage of the Type 2 SNP will rise. Algorithms such as sequential probability ratio test (SPRT) [11], Hidden Markov model (HMM) [12], and BF [1] were introduced to deduce the fetal genotype based on DCs at Type 1 and Type 2 SNPs. However, when each parent possesses a highly similar haplotype, this might lead to an insufficient number of informative SNPs for a given type. In our study, both parents bore the NM_000500.7(*CYP21A2*): EX1-7 deletion variant, and their pathogenic haplotypes were highly homologous near the pathogenic variation. For a Type 2 SNP, assuming its allele on HM2 is “*a*”, its allele on HM1 should be “*A*”, while the alleles on HF1 and HF2 are both “*a*”. Given HF1's high similarity to HM1, most alleles between HM1 and HF1 should be identical. This inconsistency resulted in only one Type 2 SNP in the studied case. Hence, it becomes challenging to accurately gauge Type 2 SNP DCs and then discern through RHDO if HM2 was inherited. Except for rare situations like uniparental diploidy (UPD), the fetus will inherit just one of HM1 or HM2. Thus, HM2's inheritance can be indirectly inferred from HM1's inheritance. This indirect method works with SPRT or HMM algorithms, but when employing the BF algorithm, the challenge arises because it requires deducing fetal genotype from the DC differences between Type 1 and Type 2, thus disallowing sole calculations based on Type 1 inheritance.

Additionally, even for SPRT and HMM algorithms, they may be affected by allelic frequency distortions when relying on a single type of SNP, particularly due to biases introduced during hybrid capture. This technical bias stems from the design of capture probes, which are typically designed based on the reference allele and thus bind more robustly to DNA templates possessing the reference allele. As a result, reference alleles are more efficiently enriched than alternative alleles, leading to skewed allele frequency measurements. Such biases are especially problematic in NIPT for monogenic disorders, where diagnosis relies on detecting subtle allele frequency imbalances in maternal plasma that reflect the underlying fetal genotypes. These imbalances exhibit significant FF dependency, where minor deviations can produce erroneous diagnostic calls [13]. Current methodologies predominantly employ solution-phase hybridization capture for SNP enrichment around pathogenic targets. However, since capture probes are designed against the reference genome, cfDNA fragments containing reference alleles demonstrate preferential hybridization efficiency. This capture bias distorts allele frequency quantification and consequently compromises recombination event detection, particularly in samples with reduced FFs [14]. In our case, out of the Type 1 loci, 23 were reference alleles (10.13%), and 204 were alternative alleles (89.87%). This imbalance implies that dosage estimates based on Type 1 SNPs could be disproportionately influenced by capture bias, potentially leading to an underestimation of fetal dosage and incorrect inference of inheritance, particularly with respect to the pathogenic haplotype HM1.

To address this limitation, our study implemented subtype stratification of Type 1–4 SNPs into reference (ref) and alternative (alt) classifications. Subsequent calculation of ref allele frequencies systematically minimized probe design-induced biases. Each SNP type was further categorized based on its allele in the respective parental haplotype into typeN_ref and typeN_alt. For example, with Type 1 SNP, if the fetus inherits HM1, the reference allele frequency for type1_ref increases by FF/2, while for type1_alt, it drops by the same magnitude (meaning the alternative allele's frequency rises). The expected reference allele frequency difference between type1_ref and type1_alt is FF. When the fetus inherits HM2, the allele frequencies for both types remain unchanged, maintaining an expected reference allele frequency difference of zero. This approach, focusing on only reference allele frequencies, effectively sidesteps allelic bias caused by DNA hybridization. With the above classification adjustments, the BF algorithm shifted its focus from measuring DC differences between Type 1 and Type 2 SNPs to computing the reference allele DC differences between type1_ref and type1_alt, ensuring compatibility even in scenarios with only one type of informative SNPs. Ultimately, according to these algorithm modifications, we successfully determined the fetus had inherited HM1, aligning with results from invasive prenatal diagnosis.

Adequate representation of informative SNPs constitutes a fundamental requirement for targeted NIPT [6]. In clinical scenarios characterized by allelic depletion—such as consanguineous unions or founder populations—conventional monogenic NIPT methodologies frequently yield no-call results or diagnostic inaccuracies. To address this limitation, we developed a haplotype refinement strategy involving subtype stratification into reference (ref) and alternative (alt) allelic variants. Subsequent quantification of relative dosage differentials between these subtypes enables robust diagnostic interpretation. This methodological advancement effectively mitigates analytical constraints stemming from haplotype-specific SNP insufficiency. This method also adapts to other haplotype-based NIPT for AR, autosomal dominant (AD), and X-linked recessive (XR) disorders. However, implementation remains contingent on the condition of enough informative SNP numbers within targeted haplotypes and an adequate number of ref and alt alleles.

## 4. Conclusions

In conclusion, we successfully implemented a monogenic NIPT in a CAH pedigree with highly similar parental pathogenic haplotypes by improving the current algorithm. Theoretically, this algorithm can be applied to resolving other situations such as consanguineous marriage and insufficient informative SNPs, further expanding the application area of haplotype-based NIPT.

## Data Availability

The raw data supporting the conclusions of this article will be made available by the authors on request.

## Nomenclature

CAH: congenital adrenal hyperplasia
NIPT: noninvasive prenatal testing
NGS: next-generation sequencing
MAF: minor allele frequency
MLPA: multiplex ligation–dependent probe amplification
SNPs: single-nucleotide polymorphisms
cfDNA: cell-free DNA
BF: Bayes factor
FF: fetal fraction
SPRT: sequential probability ratio test
HMM: hidden Markov model

## References

[1] Kong L., Li S., Zhao Z. (2021). Haplotype-Based Noninvasive Prenatal Diagnosis of 21 Families With Duchenne Muscular Dystrophy: Real-World Clinical Data in China. *Frontiers in Genetics*.

[2] Han L., Chen C., Guo F. (2020). Noninvasive Prenatal Diagnosis of Cobalamin C (cblC) Deficiency Through Target Region Sequencing of Cell-Free DNA in Maternal Plasma. *Prenatal Diagnosis*.

[3] Lun F. M., Tsui N. B., Chan K. C. (2008). Noninvasive Prenatal Diagnosis of Monogenic Diseases by Digital Size Selection and Relative Mutation Dosage on DNA in Maternal Plasma. *Proceedings of the National Academy of Sciences of the United States of America*.

[4] Lo Y. M., Chan K. C., Sun H. (2010). Maternal Plasma DNA Sequencing Reveals the Genome-Wide Genetic and Mutational Profile of the Fetus. *Science Translational Medicine*.

[5] van der Kamp H. J., Wit J. M. (2004). Neonatal Screening for Congenital Adrenal Hyperplasia. *European Journal of Endocrinology*.

[6] Kong L., Li S., Zhao Z. (2024). Exploring Factors Impacting Haplotype‐Based Noninvasive Prenatal Diagnosis for Single‐Gene Recessive Disorders. *Clinical Genetics*.

[7] Yoo S. K., Lim B. C., Byeun J. (2015). Noninvasive Prenatal Diagnosis of Duchenne Muscular Dystrophy: Comprehensive Genetic Diagnosis in Carrier, Proband, and Fetus. *Clinical Chemistry*.

[8] Wang Y., Zhu G., Li D. (2025). High Clinical Utility of Long-Read Sequencing for Precise Diagnosis of Congenital Adrenal Hyperplasia in 322 Probands. *Human Genomics*.

[9] Liu Y., Chen M., Liu J. (2022). Comprehensive Analysis of Congenital Adrenal Hyperplasia Using Long-Read Sequencing. *Clinical Chemistry*.

[10] Young E., Bowns B., Gerrish A. (2020). Clinical Service Delivery of Noninvasive Prenatal Diagnosis by Relative Haplotype Dosage for Single-Gene Disorders. *Journal of Molecular Diagnostics*.

[11] Parks M., Court S., Cleary S. (2016). Non-Invasive Prenatal Diagnosis of Duchenne and Becker Muscular Dystrophies by Relative Haplotype Dosage. *Prenatal Diagnosis*.

[12] Xu Y., Li X., Ge H. J. (2015). Haplotype-Based Approach for Noninvasive Prenatal Tests of Duchenne Muscular Dystrophy Using Cell-Free Fetal DNA in Maternal Plasma. *Genetics in Medicine*.

[13] Benn P., Zhang J., Lyons D., Xu W., Leonard S., Demko Z. (2024). Accuracy of Fetal Fraction Measurements in a Single-Nucleotide Polymorphism-Based Noninvasive Prenatal Test. *Prenatal Diagnosis*.

[14] Xu C., Li J., Chen S. (2022). Genetic Deconvolution of Fetal and Maternal Cell-Free DNA in Maternal Plasma Enables Next-Generation Non-Invasive Prenatal Screening. *Cell Discovery*.

